# Whole-Genome Sequencing of *Lentibacillus* sp. Strain JNUCC-1, Isolated from Fermented Anchovy Sauce (Myeolchi Aekjeot)

**DOI:** 10.1128/MRA.01542-19

**Published:** 2020-04-09

**Authors:** Hoo-Dhon Byun, Chang-Gu Hyun

**Affiliations:** a Department of Chemistry and Cosmetics, Jeju National University, Jeju, Republic of Korea; University of Delaware

## Abstract

*Lentibacillus* sp. strain JNUCC-1 was isolated from Korean traditionally fermented anchovy sauce. The 16S rRNA sequence of JNUCC-1 showed 95.2% and 95.1% similarity to Lentibacillus populi WD4L-1^T^ and Virgibacillus siamensis MS3-4^T^, respectively, indicating that it is a novel species. The whole-genome sequence, which contains 3,687,469 bp and 3,833 genes in 3 contigs, is reported.

## ANNOUNCEMENT

The genus *Lentibacillus* (family *Bacillaceae*, phylum *Firmicutes*) was established by Yoon et al. (1). At the time of this writing, this genus contains 16 validly named species (http://www.bacterio.net/lentibacillus.html). Bacteria of the genus *Lentibacillus* have been isolated from various samples with high concentrations of NaCl. *Lentibacillus* sp. strain JNUCC-1 was isolated from highly salted, fermented anchovy sauce made by the traditional Korean fermentation process in Chuja-do (Jeju, Republic of Korea).

The anchovy sauce sample was diluted to 10^−7^-fold with sterilized 0.85% NaCl through the serial dilution method. The sample was spread on marine agar (Difco agar 2216) containing 5% NaCl and was incubated for 5 days at 30°C under aerobic conditions. Bacteria were visually screened according to the type and size of colony and were subcultured under the same conditions until individual colonies had only a single cell type, as determined by 16S rRNA sequencing. The isolated strain, *Lentibacillus* sp. strain JNUCC-1, has been deposited in the Korean Collection for Type Cultures (KCTC) under accession no. KCTC 43912.

The genome of *Lentibacillus* sp. strain JNUCC-1 was constructed *de novo* using PacBio sequencing data. The genome sequencing was performed by ChunLab, Inc. (Seoul, Republic of Korea). Genomic DNA was obtained from cells that had been cultivated for 5 days in marine broth containing 5% NaCl using the FastDNA Spin kit for soil (MP Bio). The DNA quantity was analyzed with a Qubit 2.0 fluorometer using the Qubit double-stranded DNA (dsDNA) high-sensitivity (HS) assay kit (Invitrogen) and the Quant-iT PicoGreen dsDNA assay kit (Invitrogen). The size of the isolated DNA was measured with an Agilent 2100 Bioanalyzer using the DNA 12000 assay kit (Agilent). A SMRTbell library was prepared according to the manufacturer’s instructions (Pacific Biosciences) with no size selection. The genome sequencing was performed on the Sequel platform (Pacific Biosciences) with 2.0 sequencing chemistry and 600-min movies. *De novo* assembly of the sequencing reads was performed using the Hierarchical Genome Assembly Process version 2 (HGAP2) protocol (Pacific Biosciences). The pipeline in single-molecule real-time (SMRT) Analysis version 2.3.0 was used with default parameters. The resulting contigs were circularized using Circlator version 1.4.0 (Sanger Institute). Potential contamination in the genome assemblies was evaluated by ContEst16S.

The complete genome sequence of *Lentibacillus* sp. strain JNUCC-1 contains 3,687,469 bp, with a GC content of 43.26%, an *N*_50_ value of 2,174,790 bp, and 3 contigs with lengths of 2,174,790 bp, 25,650 bp, and 1,487,029 bp. The depth of coverage was 224.6-fold. The gene-finding and functional annotation pipelines in the EzBioCloud genome database were used. Protein-coding sequences were predicted by Prodigal version 2.6.2 (2), which showed that the genome contained 3,833 genes. In addition, 66 tRNA genes were identified by tRNAscan-SE version 1.3.1 (3), and 17 rRNA genes were identified by Rfam version 12.0 (4). The public version of the *Lentibacillus* sp. strain JNUCC-1 genome sequence deposited in GenBank was annotated using the Prokaryotic Genome Annotation Pipeline (PGAP) (5).

### Data availability.

The genome sequence of *Lentibacillus* sp. strain JNUCC-1 has been deposited in GenBank under accession no. WHOH00000000 (BioProject accession no. PRJNA577786 and BioSample accession no. SAMN13037569). The version described in this paper is the first version.
